# Chemical Characterization, Free Radical Scavenging, and Cellular Antioxidant and Anti-Inflammatory Properties of a Stilbenoid-Rich Root Extract of *Vitis vinifera*

**DOI:** 10.1155/2016/8591286

**Published:** 2015-12-14

**Authors:** Tuba Esatbeyoglu, Philipp Ewald, Yoshiaki Yasui, Haruka Yokokawa, Anika E. Wagner, Seiichi Matsugo, Peter Winterhalter, Gerald Rimbach

**Affiliations:** ^1^Institute of Human Nutrition and Food Science, University of Kiel, Hermann-Rodewald-Strasse 6, 24118 Kiel, Germany; ^2^Institute of Food Chemistry, TU Braunschweig, Schleinitzstrasse 20, 38106 Braunschweig, Germany; ^3^Division of Natural System, Graduate School of Natural Science and Technology, Kanazawa University, Kakumamachi, Kanazawa 920-1192, Japan; ^4^School of Natural System, College of Science and Technology, Kanazawa University, Kakumamachi, Kanazawa 920-1192, Japan

## Abstract

Dietary stilbenoids are receiving increasing attention due to their potential health benefits. However, most studies concerning the bioactivity of stilbenoids were conducted with pure compounds, for example, resveratrol. The aim of this study was to characterize a complex root extract of *Vitis vinifera* in terms of its free radical scavenging and cellular antioxidant and anti-inflammatory properties. HPLC-ESI-MS/MS analyses of the root extract of *Vitis vinifera* identified seven stilbenoids including two monomeric (resveratrol and piceatannol), two dimeric (trans-ɛ-viniferin and ampelopsin A), one trimeric (miyabenol C), and two tetrameric (r-2-viniferin = vitisin A and r-viniferin = vitisin B) compounds which may mediate its biological activity. Electron spin resonance and spin trapping experiments indicate that the root extract scavenged 2,2-diphenyl-1-picrylhydrazyl, hydroxyl, galvinoxyl, and superoxide free radicals. On a cellular level it was observed that the root extract of *Vitis vinifera* protects against hydrogen peroxide-induced DNA damage and induces Nrf2 and its target genes heme oxygenase-1 and *γ*-glutamylcysteine synthetase. Furthermore, the root extract could induce the antiatherogenic hepatic enzyme paraoxonase 1 and downregulate proinflammatory gene expression (interleukin 1*β*, inducible nitric oxide synthase) in macrophages. Collectively our data suggest that the root extract of *Vitis vinifera* exhibits free radical scavenging as well as cellular antioxidant and anti-inflammatory properties.

## 1. Introduction

Stilbenoids are secondary plant metabolites which are mainly present in* Vitis vinifera* L. species, the latter belonging to the plant family Vitaceae [1].* Vitis vinifera* derived stilbenoids exist as monomers, that is,* trans*-resveratrol or piceatannol, and oligomers [1] and mostly occur in the plant kingdom as* trans*-isomers (*E*) [1]. The stilbene aglycone consists of two aromatic rings linked by an ethylene bridge [1]. Beside* Vitis vinifera*, dietary sources of resveratrol (*trans*-3,5,4′-trihydroxystilbene) and its oligomers are tea, peanuts, and pistachios [1–4].  Table 1 summarizes the occurrence of different stilbenoids in cell suspension culture, berries, stems, leaves, roots, and wine of* Vitis vinifera* according to Pawlus et al. [5]. Stilbenoids exhibit antimicrobial properties and as phytoalexins they play an important role in plants defending pathogens [5].

Up to now, the majority of studies concerning stilbenoids were conducted with resveratrol as a purified standard compound. However, studies in which a complex stilbene extract of* Vitis vinifera* was applied are scarce. The use of an extract may lead to synergistic effects of the various stilbenoids as far as their bioactivity is concerned. Stilbenoids are known to exhibit potential health benefits, that are, antioxidant [3, 6], anti-inflammatory [7, 8], anticancerogenic [9], antiatherogenic [10], antiviral [11], and neuroprotective properties [12].

In the current study, we investigated potential free radical scavenging and cellular antioxidant and anti-inflammatory activities of the root of* Vitis vinifera*, which may be highly enriched with various stilbenoids. A standardized ethanol extract of the root of* Vitis vinifera* purified with ethyl acetate/*n*-hexane was applied for all studies. The qualitative and quantitative stilbenoid composition was analyzed by HPLC-ESI-MS/MS and HPLC-PDA.

Plant bioactives may prevent the oxidation of lipids, proteins, and DNA either directly by free radical scavenging or indirectly by induction of endogenous antioxidant defense mechanisms. Free radical scavenging activity was monitored by ESR spectroscopy and as spin trapping and the prevention of DNA damage was determined by the Comet assay.

The redox sensitive transcription factor nuclear factor erythroid 2-related factor-2 (Nrf2) partly regulates the expression of genes encoding antioxidant enzymes. Nrf2 is bound in the cytoplasm to its inhibitor Keap1 (Kelch-like ECH-associated protein 1). When Nrf2 is activated by electrophiles, it is released from its cytosolic protein Keap1 and binds to the antioxidant response element of the DNA in the nucleus thereby regulating the transcription of target genes including *γ*-glutamylcysteine synthetase (*γ*GCS) and heme oxygenase-1 (HO-1) [13]. Nrf2 transactivation and its target genes HO-1 and *γ*GCS were determined by a reporter gene assay, real-time PCR, and Western blotting, respectively. Paraoxonase 1 (PON1) is a high-density lipoprotein (HDL) associated enzyme which is primarily synthesized in the liver [14]. PON1 prevents low-density lipoproteins (LDL) from oxidation and thereby mediats antiatherogenic effects [14]. PON1 transactivation was measured by a reporter gene assay. Biomarkers of inflammation including interleukin 1*β* (IL-1*β*) and inducible nitric oxide synthase (iNOS) were determined in cultured cells by real-time PCR.

## 2. Materials and Methods

### 2.1. Chemicals

Methanol, HPLC grade was purchased from VWR (Leuven, Belgium) and methanol, LC-MS grade was purchased from Fisher Chemical (Loughborough, UK). Acetic acid, HPLC grade was obtained from AppliChem (Darmstadt, Germany). Doubly deionized water using a Nanopure resin (Nanopure, Barnstead) was used for high-performance liquid chromatography (HPLC) analyses.

Sodium dihydrogen phosphate dihydrate and galvinoxyl radical were purchased from Wako Chemicals (Osaka, Japan). Disodium hydrogen phosphate, hypoxanthine, xanthine oxidase, and ethanol were obtained from Nacalai Tesque (Kyoto, Japan). Hydrogen peroxide (35%) was purchased from Tokyo Chemical Industry (TCI, Tokyo, Japan). 5,5-Dimethyl-1-pyrroline-*N*-oxide (DMPO) was purchased from Sigma-Aldrich (St. Louis, MO, USA (Tokyo branch)). Ultrapure water was prepared by PWE-500 (Advantec, Tokyo, Japan) for electron spin resonance spectroscopy (ESR).

Dulbecco's modified Eagle's medium high glucose (4.5 g/L) (with sodium pyruvate and L-glutamine), Dulbecco's modified Eagle's medium high glucose (4.5 g/L) (with sodium pyruvate), Dulbecco's modified Eagle's medium high glucose (4.5 g/L), fetal bovine serum, L-glutamine 200 mM (100x), penicillin/streptomycin (100x; 10,000 U/mL penicillin; 10 mg/mL streptomycin), G-418 sulfate (50 mg/mL), fetal bovine serum, and Dulbecco's phosphate-buffered saline (DPBS) without Ca and Mg were obtained from PAA (Cölbe, Germany). Neutral red and glacial acetic acid (99–100%) were purchased from Carl Roth (Karlsruhe, Germany) and ethanol (absolute) was purchased from Merck (Darmstadt, Germany). Resveratrol and dimethyl sulfoxide were obtained from Sigma (Steinheim, Germany). Lipopolysaccharide (LPS) from* Salmonella enterica* serotype Enteritidis was obtained from Sigma. Dual-luciferase reporter assay system, phRL-TK, and passive lysis buffer were purchased from Promega (Mannheim, Germany). JetPEI and peqGOLD TriFast were obtained from Peqlab Biotechnologie GmbH (Erlangen, Germany). Primers for heme oxygenase-1 (HO-1), *γ*-glutamylcysteine synthetase (*γ*GCS), and glyceraldehyde 3-phosphate dehydrogenase (GAPDH) were ordered from Eurofins Genomics (Ebersberg, Germany). SensiMix one-step kit was obtained from Bioline (Luckenwalde, Germany). Heme oxygenase-1 antibody was purchased from Stressgen (Michigan, USA), immun-star goat anti-rabbit (GAR)-HRP conjugate secondary antibody was purchased from Bio-Rad (Munich, Germany), and Pierce ECL Western blotting substrate, spectra multicolor broad range protein ladder, and Pierce BCA protein assay kit were purchased from Thermo Scientific (Rockford, USA). GAPDH antibody and donkey anti-goat IgG HRP secondary antibody were obtained from Santa Cruz Biotechnology (Heidelberg, Germany).

Vitisin grapevine root extract (*Vitis vinifera* cultivated from vines of the area Bordeaux) was kindly provided by Wolfgang Loersch (Breko, Bremen, Germany).

### 2.2. HPLC-Photodiode Array Detector (HPLC-PDA)

HPLC analysis was done according to Macke [15]. HPLC system from Jasco (Groß-Umstadt, Germany) was used which consisted of a pump (PU-2080 Plus, Intelligent HPLC Pump), degasser (DG-2080-53, 3-Line Degasser), ternary gradient unit (LG-2080-02), autosampler (Intelligent Sampler AS-2057 Plus), and PDA (MD-2010 Plus). The separation was done on a Kromasil 100-5 C18 5 *μ*m (250 mm × 4.6 mm i.d.) column (Eka Chemicals AB, Bohus, Sweden) protected with a guard column of the same material (4 mm × 4 mm). The mobile phase consisted of 1% aqueous acetic acid (A) and methanol (B). The following gradient was used for separation: 0 min 20% B, 5 min 30% B, 15 min 30% B, 18 min 37% B, 29 min 37% B, 35 min 50% B, 57 min 50% B, 58 min 100% B, 71 min 100% B, 72 min 20% B, and 75 min 20% B. The flow rate was set at 0.8 mL/min and the injection volume was 20 *µ*L. HPLC chromatograms were recorded at *λ* = 280 nm. The root extract of* Vitis vinifera* was dissolved in methanol/water (80/20, v/v). Monomeric stilbenoids in the root extract of* Vitis vinifera* were quantified as* trans*-resveratrol equivalents and oligostilbenoids as* trans*-ɛ-viniferin equivalents. The measurements were repeated five times.

### 2.3. HPLC-Electrospray Ionization-Tandem Mass Spectrometry (HPLC-ESI-MS/MS)

A HPLC analysis was performed according to Macke [15]. HPLC system from Agilent Technologies (Waldbronn, Germany) composed of a pump (1100 Series, BinPump G1312A), autosampler (1200 Series), PDA (1100 Series, DAD G1315B), and mass spectrometer (HCT Ultra; Bruker Daltonics, Bremen, Germany) was used. The ESI conditions were as follows: ion polarity: negative, scan range: 100–3000* m/z*, dry gas temperature: 330°C, dry gas flow: 10 L/min, nebulizer pressure: 50 psi, capillary voltage: 3500 V, capillary exit: −3500 V, and end plate: −500 V. HPLC separation was achieved on a Luna 3u C18 100 A 3 *μ*m (150 mm × 2.0 mm i.d.) column protected with a guard column of the same material (4 mm × 4 mm) (Phenomenex, Aschaffenburg, Germany). Mobile phases and gradient elution were as described above. The flow rate was set at 0.2 mL/min and the injection volume at 5 *µ*L. HPLC chromatograms were recorded at *λ* = 280 nm.

### 2.4. Free Radical Scavenging Activity Measured by Electron Spin Resonance Spectroscopy (ESR)

ESR and spin trapping measurements were conducted according to Esatbeyoglu et al. [16] using a JEOL JES-FR30EX free radical monitor (JEOL Ltd., Akishima, Japan). The amplitude was set at 200 for DPPH radicals, 250 for galvinoxyl, 400 for hydroxyl radicals, and 500 for superoxide radicals.

#### 2.4.1. DPPH and Galvinoxyl Radical Scavenging Experiments

To a reaction mixture containing 360 *µ*L distilled water, 500 *µ*L ethanol, 100 *µ*L 1.875 mM DPPH (in ethanol), or 100 *µ*L 375 *µ*M galvinoxyl radical (in ethanol), 40 *µ*L of the root extract of* Vitis vinifera* (1.3, 10, 13, 40, 100, and 130 mg/mL; in case of galvinoxyl radical, 1, 1.33, 2, 4, 10, and 1000 mg/mL) was added and stirred for a few seconds. After incubating the solution for 10 min (3 h for galvinoxyl), ESR spectra were recorded.

#### 2.4.2. Hydroxyl Radical Scavenging Experiments

To the reaction mixture of 25 *µ*L 200 mM DMPO, 20 *µ*L 50 mM hydrogen peroxide, 35 *µ*L distilled water, and 10 *µ*L 0.5 mM ferric chloride, 10 *µ*L of the root extract of* Vitis vinifera* (1, 1.25, 1.67, 2.5, 5, and 10 mg/mL) was added. The reaction mixture was stirred, vortexed, and put on ice for 10 sec.

#### 2.4.3. Superoxide Radical Scavenging Experiments

To the reaction mixture of 30 *µ*L 2 mM hypoxanthine, 30 *µ*L 4 M DMPO, 26 *µ*L 200 mM DPBS buffer solution (pH 7.4), and 4 *µ*L 1 U/mL xanthine oxidase, 10 *µ*L of the root extract of* Vitis vinifera* (2, 10, 12.5, 25, 50, and 1000 mg/mL) was added. The combined reaction mixture was incubated at 30°C for 1 min.

### 2.5. Cell Lines

The detailed cell culture conditions regarding the human liver hepatoma cell line Huh7, stably transfected PON1-Huh7 cells, and human colonic adenocarcinoma cell line HT-29 are described by Esatbeyoglu et al. [16].

Murine RAW264.7 macrophages (obtained from the Institute of Applied Cell Culture, Munich, Germany) were cultured in Dulbecco's modified Eagle's medium high glucose (4.5 g/L) containing sodium pyruvate and L-glutamine supplemented with 10% (v/v) fetal bovine serum, 100 U/mL penicillin, and 100 *µ*g/mL streptomycin and grown in a humidified atmosphere of 5% CO_2_ at 37°C.

For all cell culture studies, 100 mg/mL stock solutions of the root extract of* Vitis vinifera* in ethanol and resveratrol in DMSO were prepared and stored at −80°C until further use. LPS from* Salmonella enterica* serotype Enteritidis (Sigma) was dissolved in DPBS to a stock solution of 1 mg/mL and stored at −20°C until further use.

### 2.6. Cytotoxicity (Neutral Red Assay)

Cell viability was determined using the colorimetric neutral red assay [17]. PON1-Huh7, Huh7, HT-29, and RAW264.7 cells (0.15 × 10^6^ cells/well, 0.15 × 10^6^ cells/well, 0.4 × 10^6^ cells/well, and 0.08 × 10^6^ cells/well) were seeded in a 24-well plate for 24 h. The cells were treated with the root extract of* Vitis vinifera* at various concentrations (PON1-Huh7 and Huh7 1–100 *μ*g/mL; RAW264.7 1–50 *µ*g/mL) for 24 h (PON1-Huh7 for 48 h).

### 2.7. Oxidative DNA Damage (Comet Assay)

HT-29 cells were treated with 50 *µ*g/mL root extract of* Vitis vinifera* and 50 *µ*M resveratrol as positive control for 14 h at 37°C. Subsequently, cells were treated with 25 *µ*M H_2_O_2_ in DPBS for 15 min to induce DNA damage. Oxidative DNA damage in HT-29 cells was measured using the Comet assay as described earlier [16].

### 2.8. Nrf2 Transactivation (Dual-Luciferase Reporter Gene Assay)

Transient transfection and luciferase reporter gene assay for measuring Nrf2 transactivation were conducted as described elsewhere [16].

Transiently transfected Huh7 cells were incubated with the root extract of* Vitis vinifera* (1, 5, 10, 25, and 50 *µ*g/mL) and 25 *µ*M resveratrol was used as a positive control.

### 2.9. Determination of Nrf2 Target Genes Heme Oxygenase-1 (HO-1) and *γ*-Glutamylcysteine Synthetase (*γ*GCS) (RNA Isolation and Real-Time PCR)

Human Huh7 liver cells were seeded in a 6-well plate at a density of 0.9 × 10^6^ cells/well for 24 h. Subsequently, cells were treated with 1, 10, 25, and 50 *µ*g/mL root extract of* Vitis vinifera* for 6 h. Cells were washed with DPBS and RNA was isolated using peqGOLD TriFast via phenol-chloroform extraction according to manufacturer's description.

Primers for genes of human origin were designed by Primer3 software: HO-1, F: 5′-CCAGGCAGAGAATGCTGAGT-3′, R: 5′-GTAGACAGGGGCGAAGACTG-3′; *γ*GCS, F: 5′-TTTGGTCAGGGAGTTTCCAG-3′, R: 5′-TGAACAGGCCATGTCAACTG-3′; GAPDH, F: 5′-CAATGACCCCTTCATTGACC-3′, R: 5′-GATCTCGCTCCTGGAAGATG-3′. All primers were ordered from Eurofins Genomics (Ebersberg, Germany).

SensiMix one-step kit (Quantace, Berlin, Germany) was used for real-time PCR. Human GAPDH was used as housekeeping gene.

### 2.10. Inhibition of LPS-Mediated Interleukin-1*β* (IL-1*β*) and Inducible Nitric Oxide Synthase (iNOS) (RNA Isolation and Real-Time PCR)

RAW264.7 macrophages were seeded in 12-well plates at a density of 0.2 × 10^6^ cells/well for 24 h. Afterwards, cells were treated with 20 *µ*g/mL of the root extract of* Vitis vinifera* for 24 h. LPS (10 ng/mL) was added to the cells for 4 h. RNA was isolated by peqGOLD TriFast according to manufacturer's protocol. Remaining DNA was lysed using DNAse according to manufacturer's instructions (New England Biolabs, Ipswich, USA).

Primers for murine genes were designed by Primer3 software and ordered from Eurofins Genomics (Ebersberg, Germany): Interleukin-1*β* (IL-1*β*), F: 5′-CAGCTATGGCAACTGTTCCT-3′, R: 5′-CTGGATGCTCTCATCAGGAC-3′; inducible nitric oxide synthase (iNOS), F: 5′-GGCAGCCTGTGAGACCTTTG-3′, R: 5′-GCATTGGAAGTGAAGCGTTTC-3′; GAPDH, F: 5′-CCGCATCTTCTTGTGCAGT-3′, R: 5′-GGCAACAATCTCCACTTTGC-3′.

SensiMix one-step kit (Quantace, Berlin, Germany) was used for real-time PCR. Gene expression was normalized to the housekeeping gene GAPDH.

### 2.11. HO-1 Protein Levels (Western Blot Analysis)

Whole cell extracts, total protein determination, and Western blot analysis were performed according to Wagner et al. [18] and Esatbeyoglu et al. [16].

### 2.12. PON1 Transactivation (Luciferase Reporter Gene Assay)

Luciferase reporter gene assay for measuring PON1 transactivation was described in Schrader et al. [19]. PON1-Huh7 cells were seeded at a density of 0.15 × 10^6^ cells/well in 24-well plates and incubated with 1, 2.5, 5, 15, and 25 *µ*g/mL of the root extract of* Vitis vinifera* and 25 *µ*mol/L resveratrol.

### 2.13. Statistical Analyses

Data obtained from cell culture experiments were expressed as means + standard error of the mean (SEM) or standard deviation (SD) of three independent experiments and compared to untreated cells (control) or LPS-stimulated control cells. HPLC analyses of stilbenes were expressed as means + standard deviation of five injections. Statistical analysis was performed by PASW Statistics Software Version 18 (IBM, Chicago, Illinois, USA). Data were tested for normality of distribution (Shapiro-Wilk test). Significant differences between groups were analyzed by Student's *t*-test. In case of not normally distributed data the non-parametric Mann-Whitney *U* test was applied. Significance was accepted at *p* < 0.05.

## 3. Results

### 3.1. Characterization and Quantification of the* Vitis vinifera* Root Extract

**Figure 1:**
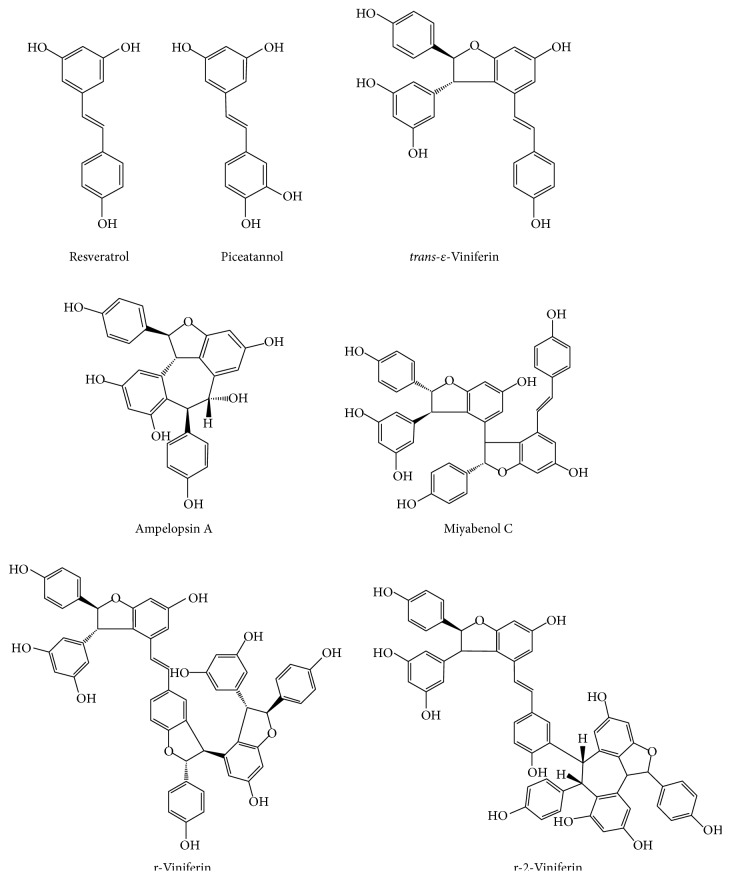
Chemical structures of monomeric and oligomeric stilbenoids of the root extract of* Vitis vinifera*.

**Figure 2:**
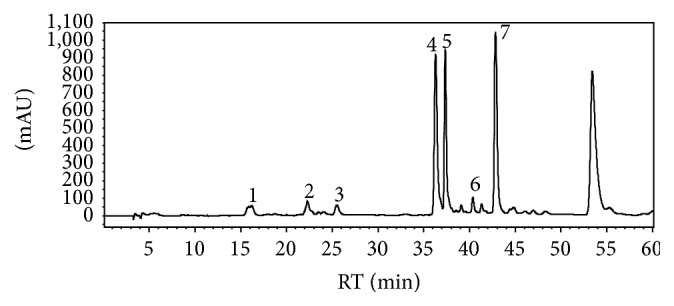
HPLC chromatogram of the root extract of* Vitis vinifera* at *λ* = 280 nm. For peak numbers, compare Table 2.

The grapevine root extract derived from* Vitis vinifera* was analyzed by HPLC-ESI-MS/MS and quantified by HPLC-PDA. HPLC-ESI-MS/MS analyses were performed using electrospray ionization operated in negative ion mode. Seven stilbenoids including two monomeric (resveratrol and piceatannol), two dimeric (*trans*-ɛ-viniferin and ampelopsin A), one trimeric (miyabenol C), and two tetrameric (r-2-viniferin = vitisin A and r-viniferin = vitisin B) were detected in the root extract of* Vitis vinifera*. Chemical structures of all detected compounds are given in Figure 1. A representative HPLC chromatogram at *λ* = 280 nm is shown in Figure 2. The main compounds in the root extract of* Vitis vinifera* were the dimer* trans*-ɛ-viniferin (125.1 g/kg), a dehydrodimer of resveratrol, and the tetramer r-2-viniferin (87.1 g/kg), composed of two resveratrol dimers (+)-ɛ-viniferin and ampelopsin B, followed by the monomer resveratrol (46.3 g/kg) (Table 2). The monomer piceatannol, the dimer ampelopsin A, the trimer miyabenol C, and the tetramer r-viniferin were present in considerably lower amounts (~4–16 g/kg) (Table 2).

### 3.2. Radical Scavenging Activity of the Root Extract of* Vitis vinifera*

**Figure 3:**
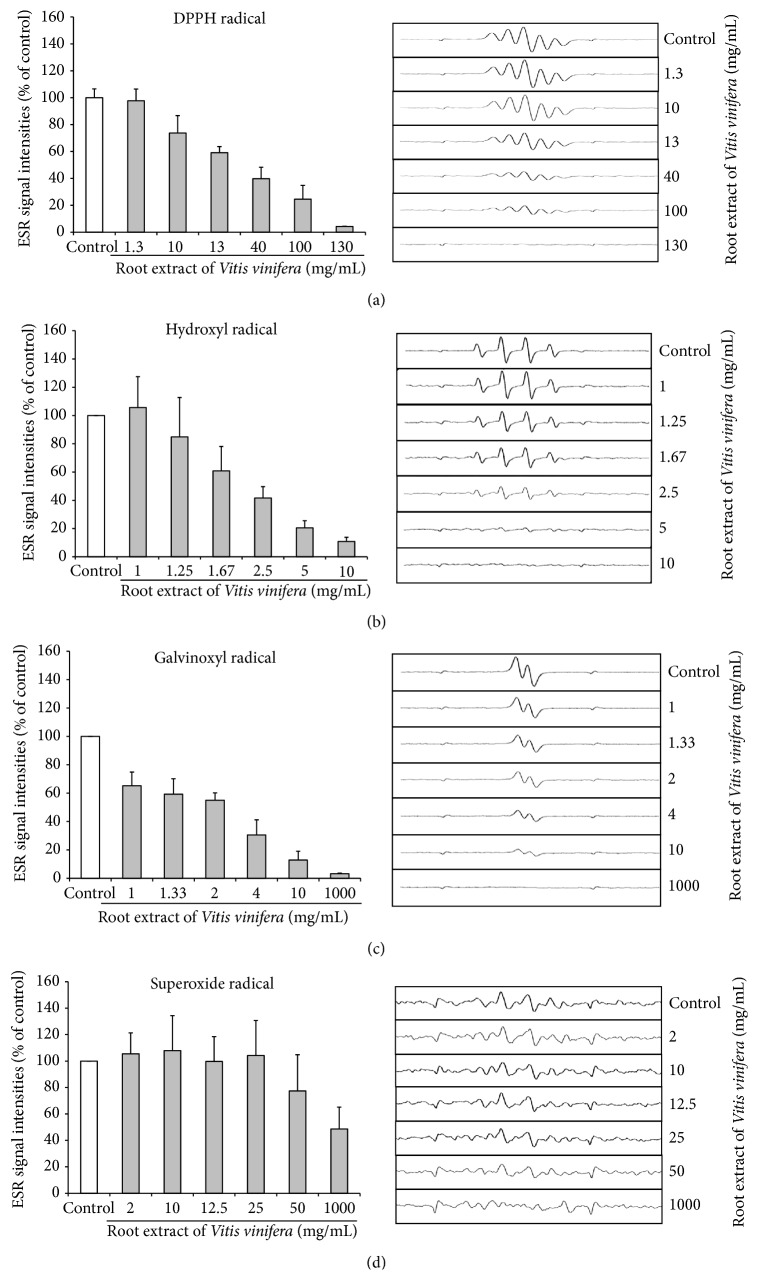
The scavenging effect of the root extract of* Vitis vinifera* on DPPH (a), hydroxyl (b), galvinoxyl (c), and superoxide free radical (d) measured by electron spin resonance spectroscopy (ESR). ESR spectra were recorded three times. Data are means + SD.

The free radical scavenging activity of the root extract of* Vitis vinifera* was determined by ESR and spin trapping analysis. The root extract of* Vitis vinifera* exhibited relatively potent free radical scavenging activity in terms of DPPH, hydroxyl, and galvinoxyl radicals and scavenged these free radicals in a dose-dependent manner (Figures 3(a)–3(c)). Superoxide radicals were scavenged at higher concentrations of the root extract (Figure 3(d)).

### 3.3. Cytotoxic Effects of the* Vitis vinifera* Root Extract in Huh7, PON1-Huh7, HT-29, and RAW264.7 Cells

Cytotoxicity measurements of the root extract of* Vitis vinifera* at different concentrations were carried out by the neutral red assay. Huh7, PON1-Huh7, HT-29, and RAW264.7 cells were treated with increasing concentrations of the root extract of* Vitis vinifera* for 24 h (PON1-Huh7 for 48 h). The root extract of* Vitis vinifera* was not cytotoxic up to a concentration of 50 *µ*g/mL in Huh7, PON1-Huh7, and HT-29 cells and up to 20 *µ*g/mL in RAW264.7 cells (data not shown). The root extract of* Vitis vinifera* was used in noncytotoxic concentrations in the subsequent cell culture experiments.

### 3.4. Prevention of Oxidative DNA Damage Induced by Hydrogen Peroxide

**Figure 4:**
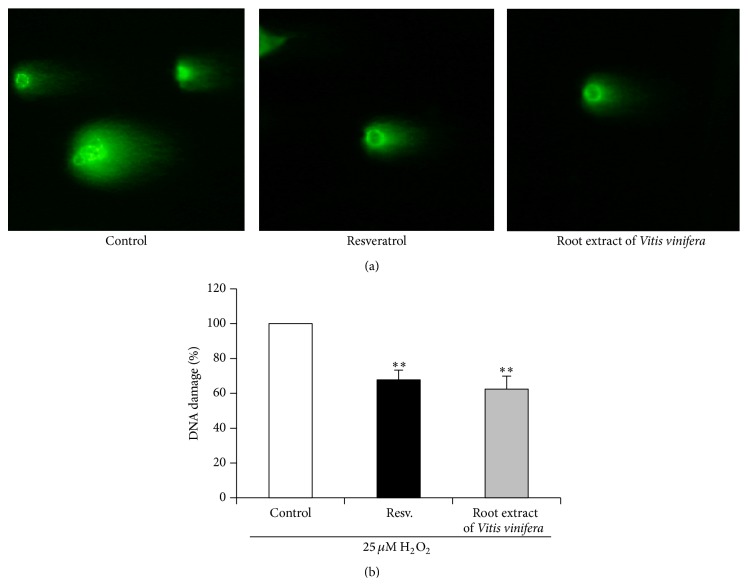
Effect of 50 *µ*g/mL root extract of* Vitis vinifera* on H_2_O_2_-induced DNA damage in HT-29 cells after 14 h of incubation. Following treatment cells were stressed with 25 *µ*M H_2_O_2_ for 15 min. Resveratrol (Resv., 50 *µ*M) was used as a positive control. DNA damage was measured by the Comet assay. The photographs represent the comet tails (a) and the inhibition of DNA damage is shown as percentage of control damage (damage of control = 100%; (b)). Each bar represents the mean of three independent experiments + SD. *∗∗* indicates significant differences compared to untreated control cells; *p* < 0.01, Student's *t*-test.

Comet assay was used to determine the effects of the root extract of* Vitis vinifera* counteracting H_2_O_2_-induced DNA damage in HT-29 cells. Treatment of HT-29 cells with 50 *µ*g/mL of root extract of* Vitis vinifera* for 14 h resulted in a moderate but significant protection of H_2_O_2_-induced DNA damage as shown in Figure 4.

### 3.5. Induction of Antioxidant Defense Mechanisms through Nrf2 Transactivation

**Figure 5:**
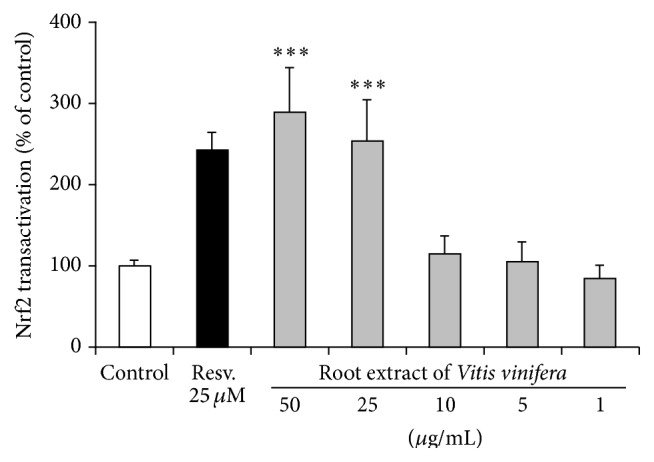
Effect of the root extract of* Vitis vinifera* on Nrf2 transactivation in transiently transfected Huh7 liver cells. Resveratrol (Resv., 25 *µ*M) was used as positive control. Data are mean + SEM of at least three experiments performed in triplicate. *∗∗∗* indicates significant differences compared to control; *p* < 0.001, Mann-Whitney *U* test.

**Figure 6:**
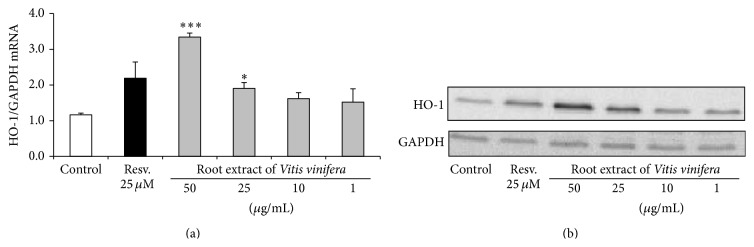
(a) HO-1 mRNA levels in Huh7 liver cells following 6 h of incubation with the root extract of* Vitis vinifera* compared to untreated control cells. Resveratrol (Resv., 25 *µ*M) was used as positive control. Data are means + SEM of at least three experiments. *∗* indicates significant differences compared to untreated control; *p* < 0.05, Student's *t*-test, and *∗∗∗* indicates significant differences compared to untreated control cells; *p* < 0.001. (b) Western blotting of HO-1 in Huh7 whole cell extracts following 24 h of incubation with the root extract of* Vitis vinifera*. Resveratrol (Resv., 25 *µ*M) was used as positive control and GAPDH was used as loading control. One representative Western blot out of three is shown.

**Figure 7:**
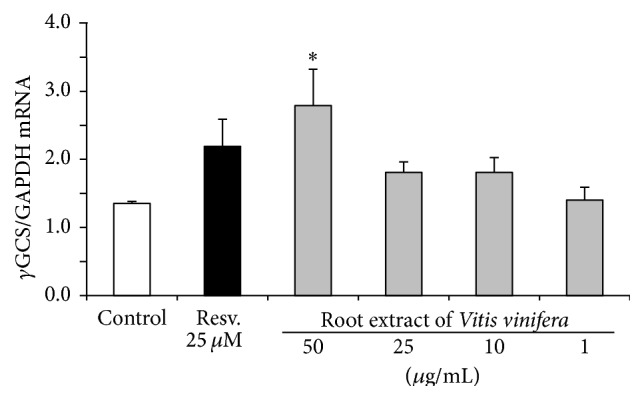
*γ*GCS mRNA levels in Huh7 liver cells following 6 h of incubation with the root extract of* Vitis vinifera* compared to untreated control cells. Resveratrol (Resv., 25 *µ*M) was used as positive control. Data are means + SEM of at least three experiments. *∗* indicates significant differences compared to untreated control cells; *p* < 0.05, Student's *t*-test.

Treatment of Huh7 cells with the root extract of* Vitis vinifera* at concentrations of 25 *μ*g/mL and 50 *μ*g/mL significantly (*p* < 0.001) upregulated Nrf2 transactivation. This effect was comparable with the Nrf2 inducing activity of 25 *µ*M resveratrol (Figure 5). Moreover, mRNA and protein levels of the Nrf2 target genes HO-1 and *γ*GCS were analyzed by real-time PCR and Western blotting in human liver Huh7 cells. The root extract of* Vitis vinifera* (50 *μ*g/mL) significantly induced HO-1 both on the mRNA (*p* < 0.001) (Figure 6(a)) and protein levels (Figure 6(b)). Accordingly, a significant induction of *γ*GCS was observed at 50 *μ*g/mL root extract of* Vitis vinifera* (*p* < 0.05; Figure 7).

### 3.6. PON1 Transactivation

**Figure 8:**
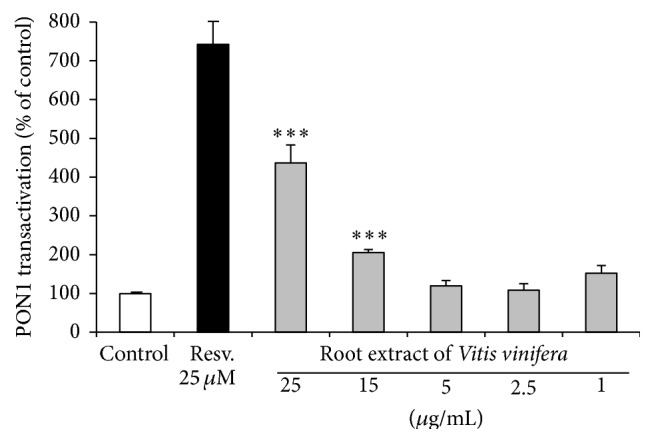
Effects of the root extract of* Vitis vinifera* on PON1 transactivation in stably transfected PON1-Huh7 cells. Resveratrol (Resv., 25 *µ*M) was used as positive control. Data are means + SEM of at least three independent experiments performed in triplicate. *∗∗∗* indicates significant differences compared to control; *p* < 0.001, Mann-Whitney *U* test.

Under the conditions investigated, luciferase reporter gene activity of stably transfected PON1-Huh7 cells was significantly (*p* < 0.001) induced by the root extract of* Vitis vinifera* in a dose-dependent manner (Figure 8).

### 3.7. Inhibition of Proinflammatory Biomarkers like IL-1*β* and iNOS due to* Vitis vinifera* Root Extract

**Figure 9:**
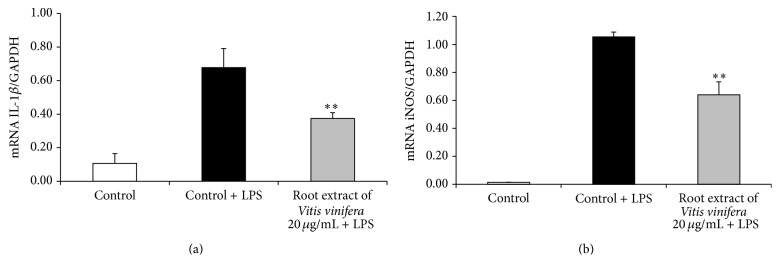
Effect of the root extract of* Vitis vinifera* on the inflammatory biomarkers IL-1*β* (a) and iNOS (b) in murine macrophages. RAW264.7 murine macrophages were incubated with the root extract of* Vitis vinifera* (20 *µ*M) for 24 h and stimulated with 10 ng/mL lipopolysaccharide (LPS) for 4 h. mRNA levels of IL-1*β* and iNOS were examined with real-time PCR. Each bar represents the mean (SEM) of at least three independent experiments measured in duplicate. *∗∗* indicates significant differences compared to stimulated control; *p* < 0.01, Mann-Whitney *U* test (a) and Student's *t*-test (b).

Furthermore, the root extract of* Vitis vinifera* (20 *μ*g/mL) significantly decreased the NF-*κ*B target genes IL-1*β* (Figure 9(a)) and iNOS (Figure 9(b)) on the mRNA level in LPS-stimulated murine RAW264.7 macrophages.

## 4. Discussion

Stilbenoids are currently receiving increasing attention due to their potential health benefits [1, 6, 7, 10–12, 20]. In this study, we combined ESR and spin trapping measurements with cellular assays in order to determine the free radical scavenging and antioxidant and anti-inflammatory properties of a root extract of* Vitis vinifera*.

Our analyses indicate that the root extract of* Vitis vinifera* contained substantial amounts of dimeric and oligomeric stilbenoids including the dimer* trans*-ɛ-viniferin and the tetramer r-2-viniferin which may have contributed to its free radical scavenging properties. The free radical scavenging activity of stilbenoids seems to be partly related to proton abstraction as previously reported [21, 22].

Since the root extract contained a portfolio of various stilbenoids, these compounds may interact synergistically thereby exhibiting free radical scavenging and antioxidant activity [23].

On a cellular level, free radicals are inactivated by endogenous antioxidant and stress response mechanisms. We found that root extract of* Vitis vinifera* exhibited HO-1 and *γ*GCS inducing activity which is most likely due to Nrf2 activation. Oxidized LDL plays a central role in atherogenesis [24]. Stilbenoids, such as resveratrol, have been shown to prevent copper mediated LDL oxidation* in vitro* through free radical scavenging activity [10]. Alternatively, our data indicate that a stilbenoid-rich extract may prevent LDL oxidation via cell signaling due to PON1 induction. Thus, stilbenoids may exhibit antiatherogenic properties due to both free radical scavenging and induction of antioxidant defense mechanisms. Interestingly, HO-1, *γ*GCS, and PON1 decrease with age [25]. Thus, it is tempting to speculate that our root extract may counteract an aging phenotype which warrants further investigations in appropriate* in vivo* models such as laboratory rodents. Furthermore, other age-related molecular targets including sirtuins [25, 26] and FOXO [26] as well as autophagy related pathways [27] should be taken into account since they have been reported to be modulated by resveratrol in cultured cells and various model organisms.

Recent studies suggest cross talk between Nrf2 and proinflammatory gene expression. Nrf2 counteracts inflammatory processes by downregulating NF-*κ*B [28, 29]. In the present study, the root extract of* Vitis vinifera* significantly decreased the expression of the NF-*κ*B target genes IL-1*β* and iNOS in murine macrophages suggesting anti-inflammatory activity. These anti-inflammatory properties of the* Vitis vinifera* root extract and other related plant extracts may be beneficial in pathologies characterized by an overproduction of nitric oxide and inflammatory cytokines [30]. Additionally, other biological properties of the root extract including its effect on platelet aggregation [31], smooth muscle cell proliferation [32], cellular adhesion [33], and vasodilation [34] should be taken into consideration.

## 5. Summary and Conclusion

In this study, seven stilbenoids including resveratrol, piceatannol,* trans*-ɛ-viniferin, ampelopsin A, miyabenol C, r-2-viniferin = vitisin A, and r-viniferin = vitisin B were identified in the root extract of* Vitis vinifera *by HPLC-PDA. The root extract of* Vitis vinifera *scavenged DPPH, hydroxyl, galvinoxyl, and superoxide free radicals. Accordingly, a protection against hydrogen peroxide-induced DNA damage was observed in cultured cells. Furthermore, Nrf2 and its target genes HO-1 and *γ*-GCS as well as PON1 were induced by* Vitis vinifera* root extract. Moreover, the root extract downregulated proinflammatory gene expression including IL-1*β* and iNOS in cultured macrophages. To sum up, our results suggest free radical scavenging and cellular antioxidant and anti-inflammatory activities of the* Vitis vinifera* root extract* in vitro*. However, little is known about the bioavailability, metabolism, and bioactivity of root-derived stilbenoids* in vivo*. Therefore, future studies should address the question to which extent stilbenoids from the roots of* Vitis vinifera* are bioavailable and may exhibit potential health benefits in humans.

In addition, both the food industry and the consumer exhibit an increasing demand for natural antioxidants [35]. Thus, further studies are needed to elucidate to which extent the stilbenoid-rich* Vitis vinifera* root extracts could prevent oxidation processes, such as lipid peroxidation, in the food matrix. Additionally, it needs to be established whether the present* Vitis vinifera* root extract may be used as a nutraceutical in functional foods.

## Figures and Tables

**Table 1:** Stilbenoids in cell suspension culture, berries, stems, leaves, roots, and wine of *Vitis vinifera* according to Pawlus et al. [5].

Plant part	Stilbenoids
Cell suspension culture	*Monomer*: *E-/Z*-Astringin, E-/*Z*-Piceid, E-/*Z*-Resveratrol, *E-*/*Z*-Resveratrol-3,4′-*O*-*β*-diglucoside, *E-*/*Z*-Resveratrol-3,5-*O*-*β*-diglucoside, *Z*-Resveratrol-3,5,4′-*O*-*β*-triglucoside, *E*-/*Z*-Resveratroloside *Dimer*: Pallidol, *E-δ*-Viniferin, *E-δ*-Viniferin-11-*O*-*β*-D-glucopyranoside, *E-δ*-Viniferin-11′-*O*-*β*-D-glucopyranoside

Berries	*Monomer*: *E*-Piceatannol, E-/*Z*-Piceid, *E*-Pterostilbene,* E*-Resveratrol

Stems	*Monomer*: *E*-Piceatannol, E-/*Z*-Piceid, E-/*Z*-Resveratrol, *E*-Resveratrol-2-*C*-glucoside *Dimer*: (+)-Ampelopsin A and F, (−)-Malibatol A, Pallidol, Scirpusin A, Viniferifuran, (+)-*E*-*ε*-Viniferin, *E*-*ε*-Viniferin *Trimer*: *E-trans*-Miyabenol C, (+)-Viniferol D *Tetramer*: Hopeaphenol, Isohopeaphenol, (+)-Viniferol A, B and C, (+)-Vitisifuran A and B, Vitisin A, *E*-Vitisin B and C

Leaves	*Monomer*: *E*-Piceid, *E*-Pterostilbene, E-/*Z*-Resveratrol *Dimer*: Ampelopsin D, Pallidol, Quadrangularin A, *E-δ*-Viniferin, *Z*-*ε*-Viniferin, (+)-*E*-*ε*-Viniferin, *E*-*ε*-Viniferin, *E*-/*Z*-*ω*-Viniferin *Trimer*: *E-*/*Z-trans*-Miyabenol C, *E-cis*-Miyabenol C, *α*-Viniferin *Tetramer*: Ampelopsin H, Hopeaphenol, Isohopeaphenol, Vaticanol C isomer

Roots	*Dimer*: (+)-Viniferether A and B, *E*-*ε*-Viniferin *Trimer*: Gnetin H *Tetramer*: Hopeaphenol, (+)-Viniferol E, *E*-Vitisin B

Wine^a^	*Monomer*: *E-/Z*-Astringin, *E*-Piceatannol, E-/*Z*-Piceid, E-/*Z*-Resveratrol, *E*-Resveratrol-2-*C*-glucoside, 2,4,6-Trihydroxyphenanthrene-2-*O*-glucoside *Dimer*: Pallidol, Pallidol-3,3′′-diglucoside, Pallidol-3-*O*-glucoside, Parthenocissin A, *E-δ*-Viniferin, *Z*-*ε*-Viniferin, *E*-*ε*-Viniferin, *E-*/*Z*-*ε*-Viniferin-diglucoside *Tetramer*: Hopeaphenol

^a^Not distinguished between red wine and white wine.

**Table 2:** Quantification of monomeric stilbenoids in the root extract of *Vitis vinifera* as *trans*-resveratrol equivalents and oligostilbenoids as *trans*-*ε*-viniferin equivalents by HPLC-PDA at *λ* = 280 nm (*n* = 5).

Peak	Compound	Retention time (*t*_*R*_) [min]	Molecular ion [M−H]^−^ *m/z*	Fragment ions *m/z*	Content [g/kg]	SD [g/kg]
1	Ampelopsin A	16.2	469	451, 363	15.6	0.41
2	Piceatannol	22.3	243	225, 201, 181, 175, 159	4.20	0.34
3	r-Viniferin (Vitisin B)	25.5	905	887, 799, 705, 675, 545, 451, 359	11.1	0.35
4	Resveratrol	36.2	227	212, 185, 159, 141, 107	46.3	0.85
5	r-2-Viniferin (Vitisin A)	37.3	905	887, 811, 705, 675, 545, 451, 359	87.1	1.31
6	Miyabenol C	40.4	679	661, 637, 585, 479, 451, 345	12.7	1.30
7	*trans*-*ε*-Viniferin	42.8	453	435, 411, 359, 347	125.1	1.23
